# Functional recombinant MHC class II molecules and high-throughput peptide-binding assays

**DOI:** 10.1186/1745-7580-5-2

**Published:** 2009-05-05

**Authors:** Sune Justesen, Mikkel Harndahl, Kasper Lamberth, Lise-Lotte B Nielsen, Søren Buus

**Affiliations:** 1 Laboratory of Experimental Immunology, Faculty of Health Sciences, University of Copenhagen, Copenhagen, Denmark

## Abstract

**Background:**

Molecules of the class II major histocompability complex (MHC-II) specifically bind and present exogenously derived peptide epitopes to CD4+ T helper cells. The extreme polymorphism of the MHC-II hampers the complete analysis of peptide binding. It is also a significant hurdle in the generation of MHC-II molecules as reagents to study and manipulate specific T helper cell responses. Methods to generate functional MHC-II molecules recombinantly, and measure their interaction with peptides, would be highly desirable; however, no consensus methodology has yet emerged.

**Results:**

We generated α and β MHC-II chain constructs, where the membrane-spanning regions were replaced by dimerization motifs, and the C-terminal of the β chains was fused to a biotinylation signal peptide (BSP) allowing for in vivo biotinylation. These chains were produced separately as inclusion bodies in *E. coli *, extracted into urea, and purified under denaturing and non-reducing conditions using conventional column chromatography. Subsequently, diluting the two chains into a folding reaction with appropriate peptide resulted in efficient peptide-MHC-II complex formation. Several different formats of peptide-binding assay were developed including a homogeneous, non-radioactive, high-throughput (HTS) binding assay. Binding isotherms were generated allowing the affinities of interaction to be determined. The affinities of the best binders were found to be in the low nanomolar range. Recombinant MHC-II molecules and accompanying HTS peptide-binding assay were successfully developed for nine different MHC-II molecules including the DPA1*0103/DPB1*0401 (DP401) and DQA1*0501/DQB1*0201, where both α and β chains are polymorphic, illustrating the advantages of producing the two chains separately.

**Conclusion:**

We have successfully developed versatile MHC-II resources, which may assist in the generation of MHC class II -wide reagents, data, and tools.

## Background

MHC molecules control the specificity of the adaptive immune system. They select peptides from any available protein in the body – be it from a foreign invader, a transformed cell, or from the protein metabolism of healthy cells – and present the selected peptides to circulating T cells for approval, or rejection, by the immune system. Two classes of MHC molecules exists, class I and II, differing with respect to function, cellular distribution and molecular composition.

In general, MHC class I molecules select peptides derived from cytosolic proteins and present them to cytotoxic T cells (CTL's) thereby endowing the immune system with the ability to examine the cellular integrity of our cells and respond to any perceived intracellular threat. Serving this function, MHC class I molecules are expressed on virtually all nucleated cells. Structurally, MHC class I molecules consist of a three-domain transmembrane heavy chain non-covalently associated with a light chain. The two outer domains of the heavy chain form a peptide-binding cleft. Bound peptides are deeply embedded in the MHC and both ends of the cleft are closed. This tends to restrict binding to peptides of limited length (e.g. 8–12 amino acids).

In contrast, MHC class II molecules select peptides derived from endocytosed proteins and present them to T helper cells (Th) thereby allowing the immune system to examine the extracellular space for the presence of protein-based pathogens. MHC class II molecules are expressed on cells involved in orchestrating immune responses; typically cells of the immune system itself (professional antigen presenting cells and activated T cells) and on activated tissues such as endothelia etc. MHC-II molecules consist of two transmembrane chains each with two domains. Despite this difference in how the chains and domains of the molecules of the two MHC classes are organized, there is a striking similarity in the structural features of the two classes of MHC molecules. The outer domains of MHC class II composed of the α_1_/β_1 _heavy chain domains forming a peptide-binding cleft, which in many respects resemble that of the outer domains of the MHC class I composed of the α_1_/α_2 _heavy chain domains. The peptide-binding cleft of MHC class II differs from that of MHC class I primarily in being open at both ends allowing peptides to extend out of the cleft. In general, MHC class II can accommodate longer peptides than MHC class I.

In most vertebrates, the MHC region is polygenic and extremely polymorphic. Thus, the specificities of peptide selection and presentation vary from individual to individual effectively reducing the risk of population-wide pathogen escape. In the human, MHC class II encompasses three isotypes, HLA-DR, -DQ and -DP. The numbers of registered human class II α and β heavy chain proteins are currently (as of January 2009): 2 and 556 for DRα and β, respectively; 25 and 69 for DQα and β, respectively; and 16 and 115 for DPα and β, respectively. For DR, the polymorphism of the peptide-binding cleft is determined solely by the β chain. For DQ and DP, pairing of α and β chains determine the polymorphism of the peptide-binding cleft. This potentially lead to as many as 1725 different DQ specificities, and 1840 different DP specificities,.

Here, we have used a disulphide-assisted refolding principle and dimerizing modules to assemble soluble, functionally empty, MHC-II heterodimers from α and β chain proteins produced independently in *E. coli *. For all nine MHC-II molecules examined here (six HLA-DR, one HLA-DP, one HLA-DQ, and one mouse I-E^d^), we successfully managed to generate functional recombinant MHC-II molecules showing specific peptide binding. Refolding was quite efficient; in some cases yields of more than 20% were obtained. Efficient *in vivo *biotinylation enabled streptavidin-based peptide-MHC-II interaction assays including several sensitive and high-throughput binding assays. These approaches to generate MHC class II molecules, and analyze their function, might meet the challenge of addressing the polymorphism of the MHC class II system.

## Methods

### Cloning of HLA constructs

All genes were generated synthetically by consecutive overlap extension PCR reactions and QuikChange mutations, or purchased from GenScript, cloned into the pET28a+ (kanamycin resistance, IPTG induction) vector and transformed into DH5α *E. coli *cells using standard molecular biology techniques. The intended DNA sequences were verified by DNA sequencing (ABI3100, Perkin Elmer). Plasmids were purified and transformed into BL21(DE3) *E. Coli *cells for protein production. To allow for in vivo biotinylation, BL21(DE3) were co-transformed with a pASYC (chloramphenicol resistance, IPTG inducible) vector with the gene encoding the BirA biotinylation holoenzyme. Clones, which produced the fusion product upon induction with IPTG, were identified and used for large-scale expression. The full length sequences of produced protein constructs can be seen in additional file 1, Figure 1.

### Expression of HLA class II α and β chain proteins in E coli inclusion bodies

All α chains were produced in shaker bottles using an auto-induction method described by Studier [1].

All β chains were expressed in a fermentor using IPTG induction as previously described [2] (except for DPB1*0401, which was produced by auto-induction). Briefly, cells were expanded overnight and used to seed a 2.5 L Labfors^® ^fermentor. Cells were grown at 37°C to an OD of 25. The temperature was then raised to 42°C and IPTG added to a concentration of 1 mM. For *in vivo *biotinylation 0.5 mM d-Biotin was added at the time of induction. After 3 hours, cells were processed at 2.3 kBar in a cell disrupter (basic Z, Constant Systems Ltd Daventry, UK). Using centrifugation (Sorvall RC6, 17,000 g, 30 min, 4°C), the inclusion body pellet was washed twice in 0.5% NP40, 0.1% DOC in PBS.

### Purification of denatured HLA α and β chain proteins

The washed pellet was dissolved overnight in 200 ml 8 M Urea, 25 mM Tris, pH 8, and any remaining DNA was precipitated with streptomycin sulphate (10 g/L). After centrifugation (Sorvall RC6, 17,000 g, 30 min, 4°C), the denatured protein solution was applied to an 800 ml Q Sepharose Fast Flow column. The column was washed with 8 M Urea, 25 mM Tris, pH 8 (Buffer A), and eluted with a two-step gradient (0–30% in 3 column volumes (CV), 30–100% in 1 CV of buffer B (A + 1 M NaCl). Fractions containing proteins of interest, as determined by SDS-PAGE, were pooled and concentrated to 100 ml using 10 kDa cut-off tangential ultrafiltration (Vivaflow 200, Vivascience AG, Göttingen, Germany). The concentrate was applied to a 3.5 L Superdex 200PG gel filtration column (GE Healthcare) and eluted with 8 M Urea, 25 mM Tris, 150 mM NaCl, pH 8. Fractions containing denatured monomer were pooled, frozen at -20°C, and used for further experiments.

### Radiolabelling peptide with ^125^I

For radio-iodination purposes the influenza hemagglutinin peptide, HA_306–318 _(PKYVKQNTLKLAT), was extended N-terminally with a tyrosine generating (Y)HA_306–318_. The peptide was ^125^I iodinated using a chloramine method [3] to a specific activity of approximately 75 Ci/mmol corresponding to 500 cpm/μl at a final working concentration of 3 nM. We noted that only 30–40% of this particular reference peptide could bind regardless of how much MHC was added. We speculated that iodination of the tyrosine in the first anchor position of the HA_306–318 _peptide might influence binding. Indeed, changing this tyrosine to phenylalanine, another accepted first anchor residue, increased the relative amount of radioactivity that could be incorporated into MHC complexes (data not shown).

### MHC class II binding of radiolabelled peptide measured by gel filtration

Equimolar concentrations of denatured DRA*0101_1–191 _and DRB1*0101_1–198 _chains (4 μM each in 8 M urea) were diluted 50 times to a final concentration of 80 nM MHC. The refolding buffer consisted of 25% v/v glycerol, 50 mM Tris, pH8, PMSF (11.4 μg/ml), pepstatin (3.3 μg/ml), TLCK (3.3 μg/ml) and TPCK (3.3 μg/ml) – and in some cases (see below) further additives. Just before use, the refolding buffer was supplemented with 3 nM ^125^I labeled (Y)HA_306–318 _peptide. The reaction mixture was incubated for 24 h at 18°C. After incubation, free and MHC-bound ^125^I labeled peptide was separated by gradient centrifugation spun column chromatography as previously described [4], and counted by gamma spectrometry (Packard Cobra 5010). The fraction of bound peptide was calculated as (peptide bound/(peptide bound + peptide free)).

### Optimizing refolding and peptide binding conditions

Various buffer compositions were tested in an attempt to optimize refolding and peptide binding: Glycerol (range 0 to 25% v/v), Arginine (0 to 500 mM), Urea (0 to 1000 mM), Sucrose (0 to 500 mM), Sodium Chloride (0 to 500 mM), N-Laurylsarcosin (0 to 1,5% w/w), Dextran (0 to 0.5% w/w), Pluronic acid F68 (0 to 0.5% w/w), Deoxycholate (DOC) (0 to 0.5% w/w), NP40 (0 to 0.5% w/w), PEG6000 (0 to 0.5% w/w), PEG20000 (0 to 0.5% w/w), beta octylglycoside (0 to 0.5% w/w), and Tween20 (0 to 0.5% w/w). Similarly, various pH conditions were tested using a refolding buffer composed of protease inhibitors (as above), 25% v/v Glycerol, and 50 mM Tris/Morpholine Ethane Sulphonic acid (MES) adjusted to pH values between 5 and 10. After 24 h incubation at 18°C, the samples were analyzed by spun column chromatography (as above).

Time and temperature conditions were tested using a refolding buffer (as above) adjusted to pH 8. Aliquots were distributed into PCR tubes and incubated at 10, 20 or 30°C with temperatures controlled by a PCR thermocycler. At different time points, tubes were removed from the thermocycler and stored at -20°C until analyzed by spun column chromatography (the high glycerol content of the buffer allowed this kind of cryopreservation).

Unless otherwise stated, the following optimal MHC class II refolding and peptide binding conditions were subsequently chosen: a refolding buffer composed of protease inhibitors (as above), 0.01% Pluriol F68, 25% v/v Glycerol, and 50 mM Tris/Citrate pH 6–7.5, and reaction mixtures were incubated for 24 h at 18°C.

### Refolding and purification of MHC class II standards

2 ml of 10 μM denatured MHC II α and β chain (DR1: DRA*0101_1–191_/DRB1*0101_1–198_(C30S); DR2a: DRA*0101_1–181_/DRB5*0101_1–190_; DR4: DRA*0101_1–181_/DRB1*0401_1–190_) was diluted drop wise into 60 ml refolding buffer pH 7.5 containing 2 μM HA_306-318H6 _(PKYVKQNTLKLATHHHHHH). After 48 h incubation at 18°C, the refolding mixture was loaded onto a 6 ml Ni^2+ ^charged IDA column. The column was washed with PBS (Buffer A) until the UV280 signal reached baseline followed by a two segment gradient (0–20% in 5 CV, and 20–100% B in 2 CV) with buffer B (PBS supplemented with 250 mM Imidazole). Fractions were collected and analyzed by reducing SDS-PAGE.

### MHC class II binding measured by ELISA

Monoclonal murine antibodies LB3.1[5,6] (mouse anti-DR α chain), D1.12 [7] (anti-DR), L243 [8] (anti-DR α chain) G8 [9] (anti-DR), 9.3F10 [6,10] (anti-DR, DQ), 2.06 [11] (anti-DR), B7/21 [6] (pan specific-DP), and 14.4.4S [12] (anti I-E^d^) were purified from culture supernatants (L243 and 14.4.4S), or ascites, by anion exchange or protein A chromatography.

Appropriate MHC class II standards were serially diluted in PBS and added to a Streptavidin plate (NUNC, Denmark), which had been blocked in 5% skim milk powder (SMP). After 1 h incubation at RT, the plate was washed 3 times in PBS with 0,01% Tween-20, and monoclonal anti-MHC class II antibodies were added (50 μl/well of a 10 μg/ml dilution in PBS with 2% SMP). After 1 h incubation at RT, the plate was washed 3 times in PBS with 0.01% Tween-20, and HRP conjugated goat anti-mouse antisera (A9917, Sigma Aldrich) was added (50 μl/well of a 1/12,000 dilution in PBS with 2% SMP). After 1 h incubation at RT, the plate was washed 3 times in PBS with 0.01% Tween-20, and 3,3',5,5'-tetrametylbenzidin (TMB) "One-Step Substrate" (DAKO) was added (50 μl/well). After 30 min incubation at RT in the dark, sulfuric acid (50 μl/well of a 0.3 M solution) was added to stop the color reaction, and the plates were read at 450 nm in a Powerwave microplate spectrometer (Bio-Tek instruments).

To identify optimal concentrations of MHC class II α and β chains (DR1: DRA*0101_1–191_/DRB1*0101_1–198_(C30S); DR2a: DRA*0101_1–181_/DRB5*0101_1–190_; DR4: DRA*0101_1–181_/DRB1*0401_1–190_), six titrations of α chains, and eight of β chains, were diluted in 8 M Urea, 25 mM Tris, pH 8; and the two titration series were combined to create a checkerboard of 48 different combinations of α/β concentrations. These were further diluted 33 times into a refolding buffer pH 6–8 with, or without, 2 μM HA_306–318_. After 24 h incubation at 18°C, the plates were developed in an ELISA (as described above) using appropriate anti-MHC class II monoclonal antibodies as detection antibodies. Signals and signal/noise ratios were calculated for each combination of α/β concentrations.

Using optimal α/β concentrations, denatured MHC class II α and β chains were diluted into refolding buffer with a titration of test peptide, and incubated for 48 h at 18°C. The ELISA was used to measure the resulting peptide-MHC class II complexes. The absorbance values (450 nm) were graphed vs. the concentrations of peptide offered, and the data analyzed by GraphPad Prism as described below.

### MHC class II binding measured by Luminescent Oxygen Channelling Immunoassay (LOCI)

LOCI (marketed by Perkin Elmer as AlphaScreen) is a two-bead assay system: donor beads contain a photosensitizer compound, which upon illumination with laser light at a wavelength of 680 nm converts ambient oxygen to energy-rich, short-lived singlet oxygen; and acceptor beads, which can respond to singlet oxygen with a luminescence/fluorescence cascade leading to an amplified signal in the 520–620 nm range. The oxygen released by a donor bead will only excite acceptor beads within a distance of ~200 nm, and this allows a proximity-based homogenous assay quantifying interacting biomolecules. We have recently described a LOCI-based HTS assay for measuring peptide-MHC class I interaction [13].

Here, donor beads coated with streptavidin, and acceptor beads for custom protein coupling were both purchased from PerkinElmer. Specific anti-MHC class II monoclonal antibodies were coupled to acceptors beads following the manufacturers recommendation (L243 for HLA-DR; B7/21 for HLA-DP; 9,3F10 for HLA-DQ; and 14-4-4 for I-E^d^). Peptide-MHC class II reaction mixtures were generated and incubated 48 h at 18°C as described above for the ELISA. The peptide-MHC class II reaction mixtures were then mixed with equal volumes (12–25 μl) of a solution containing streptavidin donor beads and anti-MHC-II monoclonal antibody conjugated acceptor beads (both at 10 μg/ml in PBS containing 0,1% Pluronic F68). The plates were incubated for 18 h at 18°C and then read in an ENVISION reader (Perkin Elmer). As for the ELISA, the optimal combination of α and β chain concentrations was identified in pilot experiments. Then, peptide titrations and the resulting peptide-MHC class II formations were determined in a LOCI assay calibrated with a known MHC class II standard.

A LOCI-based competitive assay was also developed. In this assay, a binding reaction was set up between a trace concentration of a biotin labeled agonist peptide (biotin attached via a PEG linker to HA_306–318_) and non-biotinylated MHC-II molecules. For many HLA-DR molecules one could use low nanomolar (*in casu *4 nM) concentrations of agonist peptide. The resulting agonist-MHC-II interactions were developed using a LOCI assay as described above. Once an agonist-MHC-II interaction assay had been established, competition assays using titrations of any test peptide of interest could be conducted.

### Peptide affinity calculations

The formation of peptide-MHC complexes was calculated from LOCI generated data, which had been calibrated using standard curves obtained with purified peptide-MHC complexes of known concentrations.

For direct binding experiments, the concentrations of peptide-MHC class II complexes formed were graphed versus the concentrations of peptide offered, and analyzed by non-linear regression (GraphPad Prism). The peptide concentration resulting in half saturation, the half maximal effective concentration (EC_50_), was estimated by fitting the experimental data to the equation Y = Bmax*X/(K_D_+X), where Y is the concentration of peptide-MHC-II complexes formed and X is the concentration of ligand (peptide) offered. The EC_50 _approximates the K_D _as long as the receptor concentration used is less than the K_D _thus avoiding ligand depletion.

For the competitive binding assay, the concentrations of peptide-MHC class II complexes formed were graphed versus the logarithm of the concentrations of inhibitory peptide used, and analyzed by non-linear regression (GraphPad Prism). The peptide concentration resulting in half inhibition, the half maximal inhibitory concentration (IC_50_), was estimated by fitting the semi-log transformed experimental data to the equation Y = Bottom + (Top-Bottom)/(1+10^(X-Log(IC_50_))), where Y is the concentration of agonist-MHC-II complexes formed, X is the logarithm of the concentration of inhibitor offered, Top is the upper plateau of the curve (i.e. without inhibitory peptide) and Bottom is the lower plateau of the curve (i.e. with saturating concentrations of the inhibitory peptide). The IC_50 _approximates the K_D _of the inhibitor peptide as long as the receptor concentration used is less than the K_D_'s of both the agonist and inhibitor peptides [14].

## Results

### Design of MHC class II α and β chains

HLA class II α and β chain polypeptide sequences were obtained from the IMGT/HLA Sequence Database. The I-E^d ^α and β chains were obtained from the Swiss-Prot database ; accessions P01904 and P01915, respectively. Gene sequences were codon-optimized for *E coli *expression and truncated to remove the transmembrane regions. Most chains were produced in two different truncations; a short and a long version of the α chain comprising amino acids 1–181 and 1–191, respectively; and a short and a long version of the β chains comprising amino acids 1–190 and 1–198, respectively (see additional file 1, Figure 4).

We have previously demonstrated how unpaired cysteines can seriously reduce functional yield [15,16]. Unpaired cysteine residues in positions 30, 47 and 123 of DRB1*0101, DQA1*0501 and DPA1*0103, respectively, were therefore mutated to serine. Position 30 of DRB1*0101 and 47 of DQA1*0501 is located in the binding groove and might affect the peptide binding specificity, albeit a cysteine to serine might be considered a conservative substitution in an MHC context. Position 123 of DPA1*0103 is located in the second domain and would not be expected to influence the specificity of peptide binding.

Initially DQA1*0501 expressed poorly in bacteria, however N-terminal fusion of the natural histidine affinity tag (HAT) increased expression.

To assist in α/β chain assembly, a pentaglycine linker followed by the fos and jun leucine zipper was added to the C-terminal of the α and β chain, respectively. To allow for specific enzymatic biotinylation, the jun leucine zipper of the β chain was further extended by a triglycine linker and a biotinylation substrate peptide [17]. Using the *E. coli *GrpE chaperone to direct dimerization, an alternative α/β chain assembly system was successfully developed (see additional file 1).

In all cases, the β chains were produced with a C-terminally added biotinylation substrate peptide (BSP) allowing *in vivo *biotinylation using co-induced BirA holoenzyme to couple d-biotin specifically to the BSP sequence [18].

The resulting polypeptide sequences are shown in additional file 1, Figure 1.

### Expression and Purification of MHC class II α and β chains

**Figure 1:**
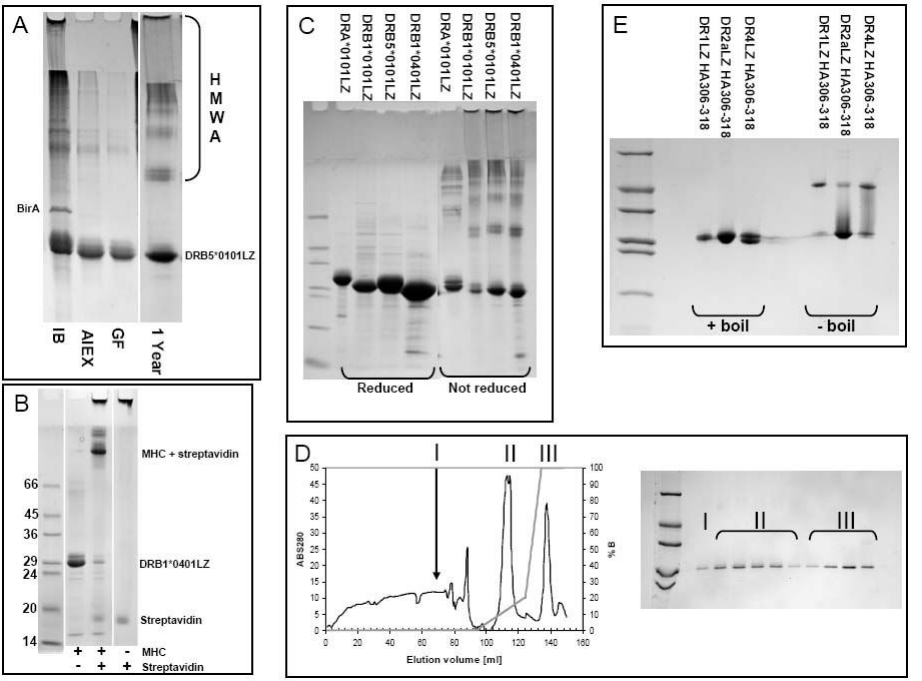
**Purification of MHC II and refolding of complexes**. (A) Non reducing gel showing purification of urea denatured DRB5*0101. IB: urea denatured inclusion bodies after fermentation. AIEX: Purity after ion-exchange on Q-Sepharose FastFlow. GF: Purity after gel filtration on Superdex200, this is the final product from the purification. Though pure, the MHC band seems to consist of several sub bands each representing an oxidative isomer of the molecule. 1 Year: Same product after 1 year at -20°C. Note the formation of higher molecular weight aggregates (HMWA), and a more concentrated MHC band. (B) Biotinylation test, gel shift assay of DRB1*0401. Without streptavidin MHCbiotin travels as a monomer, addition of an excess of streptavidin causes nearly all MHCbiotin to shift to a position corresponding to a 1:1 complex with streptavidin, without MHCbiotin streptavidin travels as a monomer. In most cases a nearly quantitative biotinylation could be achieved. (C) Reducing and non-reducing SDS-PAGE of DRA*0101, DRB1*0101, DRB5*0101 and DRB1*0401. (D) IMAC purification of DR2a refolded in the presence of a histidine tagged HA_306-318H6 _peptide and eluted by an imidazole gradient. Fractions were collected and analysed by reducing SDS-PAGE (samples boiled). Peak I: Run through Peak II: empty α and β chain binding with moderate affinity to Ni^2+ ^column Peak III: Elution of MHC II complex by imidazole. (E) Non-reducing SDS-PAGE analysis of DR1, DR2a and DR4 complexes refolded and purified as in D. Note the SDS stability of DR1 and DR4. The final yields were 12, 23 and 14%, respectively.

After induction, the recombinant proteins were contained in bacterial inclusion bodies. Inclusion bodies were processed by high-pressure cell disruption, harvested and washed by centrifugation. The recombinant proteins were extracted from the inclusion bodies into 8 M Urea and purified by Q Sepharose anion exchange chromatograhy followed by Superdex 200PG gel filtration chromatography. Pure preparations were obtained as demonstrated by non-reducing SDS-PAGE (exemplified for DRB5*0101, Figure 1A). The yields were typically 50–70 g/L for autoinduction and 80–120 g/L for IPTG induction of pure α and β chain, respectively.

Denaturing, but non-reducing, conditions were maintained throughout the extraction, purification and subsequent storage procedures. We have previously described that a large fraction of MHC class I molecules produced in this manner is correctly oxidized and refolds very efficiently [2,16,18]. Being structurally related to MHC class I, we reasoned that MHC class II molecules might also be produced in a highly active, pre-oxidized form. Indeed, non-reducing SDS-PAGE showed slightly smeared MHC bands (Figure 1A), or in some cases even distinct and closely appositional bands, which disappeared upon reduction and generated a single band (Figure 1C). Overloading the SDS-PAGE only visualized few contaminants; mostly high-molecular aggregates of MHC since they disappeared upon reduction while the staining of the MHC band increased (Figure 1C). Storage at -20°C for a year gave rise to modest formation of high-molecular weight aggregates and complexes (Figure 1A), which disappeared upon reduction. Together these data suggest that our preparations predominantly contained MHC proteins, and as expected consisted of different di-sulfide isomers.

We have previously reported that *in vivo *enzymatic biotinylation can be very efficient. For a large cohort of MHC class I molecules we routinely obtained biotinylation efficiencies in excess of 90–95%. Similar high biotinylation efficiencies were obtained for MHC class II as illustrated by a SDS-PAGE gel-shift assay where an excess streptavidin led to a mobility shift of almost the entire population of monomers (Figure 1B).

### Characterization of peptide-MHC class II complexes

A high-affinity binding peptide with a hexa-histidine affinity tag was used to purify functional DRA*0101/DRB5*0101 (DR2a) heterodimers. Purified denatured DRA*0101_1–181 _and DRB5*0101_1–190 _were diluted drop-wise into a refolding buffer containing a histidine-tagged influenza peptide, HA_306-318H6_, incubated for 48 h at 18°C, and then purified by immobilized Ni^2+ ^affinity chromatography (Figure 1D). Samples were taken from each fraction, reduced and boiled, and analyzed by SDS-PAGE (Figure 1D insert). Since empty α and β chains bound with low affinity to the Ni^2+ ^column, a two-segment gradient was used to separate empty chains from complexes. The chromatogram therefore showed two elution peaks, which both contained MHC molecules (labeled II and III in Figure 1D and insert).

However, the late peak was only seen when the HA_306-318H6 _peptide was present during refolding, whereas the early peak was generated even in the absence of the peptide (data not shown). To further support that the late peak contained relevant peptide-MHC class II complexes, a non-reducing SDS-PAGE gel was performed with and without boiling the samples. It has previously been shown that many peptide-MHC class II complexes are resistant to SDS-PAGE as long as they are not boiled [19]. The hexa-histidine tagged HA_306-318H6 _peptide was used to generate and purify three different DR molecules, DR1, DR2a and DR4, as described above, and samples from the late peak subjected to boiling and SDS-PAGE. For all three DR molecules, the late peaks contained SDS-resistant, but heat sensitive, complexes (Figure 1E), whereas the early peaks did not contain SDS-resistant complexes (data not shown). We conclude that our recombinant MHC-II molecules exhibit the known peptide-dependent SDS-sensitivity of native MHC-II molecules.

We also noted that the folding efficiencies of our recombinant DR1, DR2a and DR4 molecules interacting with the Ha_306-318H6 _peptide, under the conditions of these initial experiments, were quite high: 12, 23, and 14%, respectively.

### Development of a peptide-MHC class II binding assay

**Figure 2:**
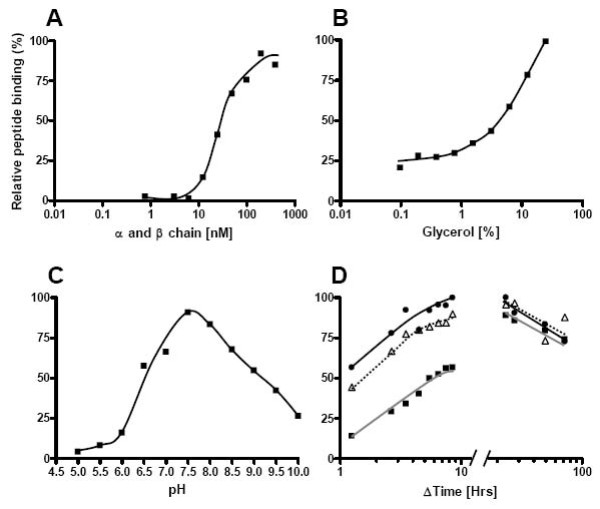
**Initial optimization of DR1 refolding**. DR1 refolding was determined using a radioactive peptide and spun column separation of free and HLA-DR1 bound peptide. Y-axis shows the relative amount of radioactive peptide bound in each experiment. (A) Titration of equimolar concentrations of urea denatured DR1 α and β chain into a refolding buffer containing 3 nM ^125^I labelled (Y)HA_306–318 _peptide. In experiments B-D, the DR1 concentration used was at 80 nM. (B) Titration of Glycerol from 25 to 0.1%(v/v). (C) Impact of pH on DR1 complex formation. (D) Kinetic of DR1 complex formation at three different temperatures (black square, grey line) 10°C, (triangle, dotted line) 20°C and (black circle, black line) 30°C.

To optimize the refolding conditions of MHC-II molecules, we initially employed a robust and easily interpretable assay involving binding of radioactively labeled peptide to MHC class II molecules. Briefly, a dose-titration of equimolar denatured DR1 α and β chains were diluted into refolding buffer containing 3 nM of a ^125^I labeled reference peptide, (Y)HA_306–318_, and incubated (24 h, 18°C). Binding was determined by gradient-centrifugation spun column gel-filtration separating bound vs. free peptide [4]. An MHC class II dose-dependent binding was observed with a half maximal binding occurring at approximately 20 nM (Figure 2A). In an attempt to optimize the binding reaction, we tested the effects of various commonly used refolding additives such as arginine, glycerol, sucrose, detergents, alcohols etc. Glycerol was found to have the a pronounced positive impact upon refolding yields (Figure 2B); no other investigated refolding additive had any significant positive effect upon refolding and many had a negative effect (additional file 1, Table 1). The positive effect of glycerol upon MHC class II folding has been noticed by others [8,20-22]. Refolding of proteins can be very pH dependent and the effect of pH was therefore also examined. The optimal pH for refolding and incorporation of radio labeled (Y)HA_306–318 _was found to be around pH 7–8 (Figure 2C). Then, the kinetics of peptide binding to *de novo *diluted recombinant MHC class II α and β chains at different temperatures were investigated. The rate of peptide complex formation was found to be highly temperature dependent (Figure 2D). Steady state bindings were reached after 8, 11 and 48 h incubation at 30, 20, and 10°C, respectively. For all temperatures, a decline in bound peptide was observed for incubation periods longer than 20–30 h. This is probably due to the stability of one or more of the assay components being compromised during long-term incubation.

**Table 1:** Affinity measurements using LOCI assay.

		**DR1GrpE^a^** **Komp^b^**	**DR2a** **Komp^b^**	**DR2a pH6**	**DR3**	**DR4**	DRB1*0813	DRB3*0301	DQA1*0501 DQB1*0201	**DP401**	**I-E^D^**	**Reference**
Designation	Sequence	IC50 (nM)	IC50 (nM)	nM	nM	nM	nM	nM	nM	nM	nM	
MBP	FLYGALLLA	3921	NB	NB	NB	NB	NB	N.D	N.D	34	NB	[26]
TERT572Y	YLFFYRKSV	4287	NB	2648	NB	1593	95	N.D	N.D	NB	322	
Flu PR8 derived	FMYSDFHFI	642	1018	637	NB	1130	NB	N.D	N.D	1	NB	[27]
Pox.o42.con A2	FLIDLAFLI	NB	4276	NB	NB	NB	NB	NB	NB	179	NB	
Pox.o42.con.im. A2	RMIAISAKV	1464	1985	1647	516	NB	267	1259	NB	NB	NB	
Brucella	WMARRHAER	NB	NB	NB	NB	NB	224	NB	NB	NB	6	
Salmonella	MMAWRMMRY	7	25	18	1709	68	28	1426	4318	NB	837	
Flu HA1194-202	IVFMWAIHH	42	529	167	NB	NB	276	NB	NB	NB	NB	
Hantaan	FMSRKLHRY	NB	2552	NB	279	NB	21		NB	NB	3	
Oxytocinase271	YEKKYFAATQFEPLAARL	5	29	6	38	6	38	33	1090	1	NB	[79] (DP401)
Thyroid peroxidase	YIDVWLGGLAENFLPY	605	691	2138	NB	NB	NB	3184	564	602	NB	[6]
No nat. source	YPFIEQEGPEFFDQE	1279	NB	NB	289	3972	NB	3228	648	NB	NB	[6]
Hepatitis B	YLCQVFADATPTGWGL	1850	NB	3703	69	18	2988	2058	1335	13	NB	[6] (DR3, DR4)
Plasmodium Falciparum	YILLKKILSSRFNQM	7	2	4	32	94	4	1	NB	1	20	[28](DR1, DR2a, DR4)
No natural source	YILFLVKMNALRRLPV	10	20	5	39	?	78	3	NB	45	582	[28] (DR1, DR2a, DR4)
Hepatitis B	VGNFTGLYSSTVPVF	3	31	102	1414	632	2892	9	635	NB	NB	
Influenza	PLKAEIAQRLEDV	50	41	65	134	2678	NB	11	NB	2091	NB	[29] (DR1, DR2a)
HIV	PIVQNIQGQMVHQ	71	852	447	471	NB	NB	23	NB	NB	NB	[30](DR1)
HepatitisB	PDRVHFASPLHVAWR	5	26	8	6	881	39	3	2807	124	NB	
Clostridium tetanus	QYIKANSKFIGITE	18	11	22	30	432	29	1	NB	56	1501	[29] (DR1, DR2a)
No nat. source	KILEPFRKYTAFTIP	10	1	16	18	1600	11	1015	1542	30	2198	[34] (DR2a)
Hepatitis B	AFSYMDDVVLGAKSV	215	3382	1721	87	26	2753	764	2039	NB	NB	
Mycobact leprea	GVTYEIDLTNKN	NB	NB	NB	4346	2548	NB	NB	NB	NB	NB	[6] (DR4)
Murine β_2_m	IQMLKNGKKIPKVEMS	1232	3	6	21	NB	73	70	NB	NB	117	[31] (I-Ed)
Lambda repressor	YLEDARRLKAIYEKKK	2999	34	87	1098	3120	136	NB	NB	NB	184	[31] (I-Ed)
6xH Lambda 2(315)	HHHHHHIEGRFAALWFRNHFVFGGGTK	4	20	5	882	11	8	1198	NB	2	26	[32]
6xH Influenza A (PR8) HA 109–119	YHHHHHHIEGRSFERFEIFPKE	325	23	9	1429	1869	181	1528	3913	1	984	[33]
CLIP 6xH	LPKPPKPVSKMRMATPLLMQALPMYHHHHHH	1	1	1	1	1	2	1	224	1	23	
CLIP	YLPKPPKPVSKMRMATPLLMQALPM	1	1	1	1	2	2	1	381	4	72	
HA306-318	YKYVKQNTLKLAT	7	2	10	13	8	11	4	NB	NB	25	Most DR

Values represent an average of 2–4 measurements. Affinity measurements higher than 5000 nM were categorized as non-binders (NB). N.D: Not determined. a: The DR1 allele used for these measurements was produced as a construct comprising amino acids 1–181 and 1–190 of α and β chain respectively fused C terminally to the dimerizing nucleotide exchange factor GrpE (see additional file 1), this construct was not biotinylated and used in the LOCI competition assay. b: Determined using competitive LOCI assay. The reference column also states the MHC alleles known to bind the peptide.

**Figure 3:**
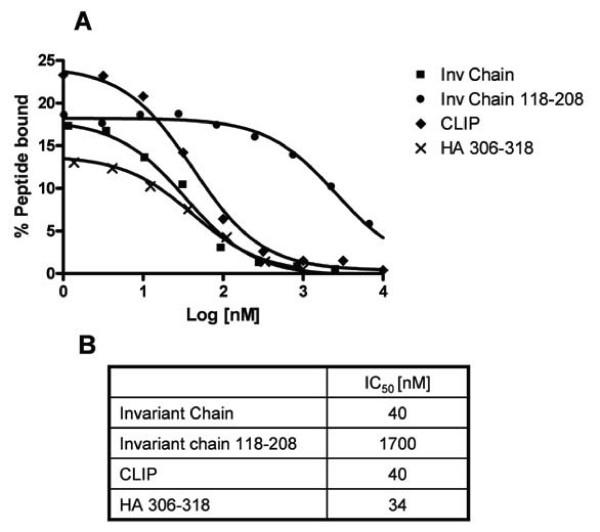
**Competitive peptide-HLA-DR1 binding assay**. Titration of peptides and proteins on DR1 using radioactive spun column assay. (A) X-axis: Log10 to peptide concentrations in nanomolar, Y-axis: percent of offered ^125^I labelled (Y)Ha_306–318 _peptide incorporated into DR1 complexes. 80 nM of urea denatured DR1 α and β chain was diluted into refolding buffer supplemented with titrations of peptide or protein and 3 nM ^125^I labelled YHa_306–318 _peptide. The following competitors were used: a recombinant version of the extracellular part of human invariant chain (positions 73–208), the CLIP peptide fragment of the invariant chain (positions 97–120), a C-terminal fragment of the invariant chain (positions 118–208), and the HA_306–318 _peptide. After 24 h of incubation, fractions were analyzed using the spun column assay and IC_50 _values (B) determined as described in Materials and Methods. All curves fitted with a regression coefficient better than 0.99. Note that the MHC concentration used (80 nM) bound approximately 70–80% of reference peptide (see Figure 2A), hence some degree of ligand depletion could be expected.

Competition experiments were performed to ascertain that the observed peptide-MHC class II bindings were saturable and specific. Increasing concentrations of competing peptide were added to a reaction involving binding of ^125^I labeled (Y)HA_306–318 _to DR1. The resulting binding was measured in the spun column assay and depicted as an inhibition curve (Figure 3). Using GraphPad Prism, non-linear fittings of the data were performed in all cases obtaining regression coefficients better than 0.99. IC_50 _values could be calculated. The HA_306–318_, the Invariant chain (positions 97–120, the CLIP peptide), and the Invariant chain (positions 73–208), were all good binders to DR1, whereas a C-terminal Invariant chain fragment devoid of CLIP (pos 118–208) was a poor binder. The fragment was previously shown to interact with empty HLA-DR1 molecules, augmenting rather than blocking binding of peptide [23].

Thus, using isolated recombinant MHC class II α and β chains it is possible to obtain specific peptide-MHC class II interaction, and measure the affinity of interaction.

Subsequent binding experiments were conducted with pre-oxidized MHC-II chains diluted into a refolding buffer containing peptide and 25% glycerol, 50 mM Tris/Citrate (or MES), a protease inhibitor cocktail, 0.01% pluronic acid F68, pH 8. This reaction mixture was incubated for 24 h at 18°C, and then analyzed for complex formation.

### Efficient refolding of pre-oxidized, denatured MHC-II proteins

**Figure 4:**
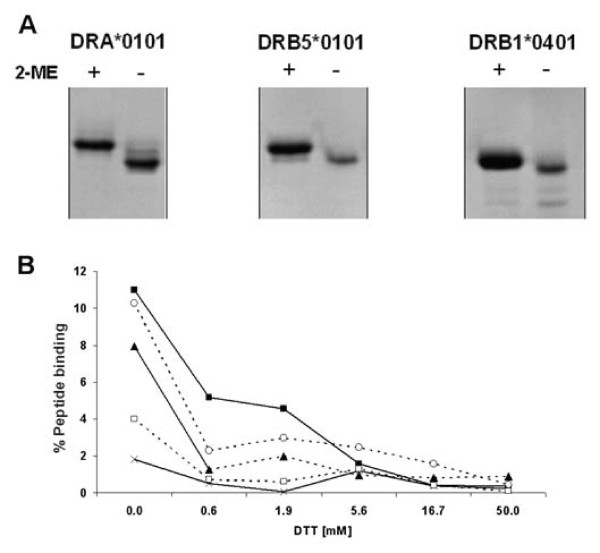
**Disulphide assisted refolding of MHC class II**. Comparison of disulphide assisted refolding with traditional oxidative refolding. (A) Gel shift assay showing that reduction of DRA*0101, DRB5*0101 and DRB1*0401 (by 2-mercaptoethanol (2-ME)) resulted in formation of a slower moving isomer, and suggesting the presence of oxidized isomers in non-reduced samples. (B) Urea denatured β chain (4 μM) was reduced in a titration of dithiotreitol (DTT) and diluted 50 times into refolding buffer pH 8 containing various redox pairs plus denatured non-reduced α chain and ^125^I labeled (Y)HA_306–318_. After 24 h incubation, the experiment was analyzed by spun column assay. X-axis: Concentration of DTT used to reduce β chain; Y-axis: percent of offered ^125^I labelled (Y)Ha_306–318 _peptide incorporated into peptide-MHC-II complexes. (black square) No redox pair (open circle) Reduced/oxidized glutathione 5/0.5 mM, (black triangle) Reduced/oxidized glutathione 2/2 mM (open square) Reduced/oxidized DTT 5/0.5 mM, (cross) Reduced/oxidized DTT 2/2 mM.

The above peptide-binding assay was used to investigate the basic premise of our approach: that pre-oxidized MHC class II molecules refold more efficiently than fully reduced MHC class II molecules. SDS-PAGE analysis of reduced vs. non-reduced α and β chain proteins clearly demonstrated that pre-oxidized species are present in the non-reduced protein preparations (Figure 4A). Preparations of denatured β chain proteins were reduced with graded concentrations of DTT, and then diluted into an excess refolding buffer containing various redox pairs as well as non-reduced denatured α chain and radiolabeled (Y)HA_306–318 _peptide, and incubated. The resulting complex formation was analyzed by spun column gel filtration as described above. Whereas the pre-oxidized species were highly active with respect to peptide binding, exposing the denatured β chain proteins to reduction with as little as 0.6 mM DTT lead to a significant loss of peptide-binding capacity, and none of the tested concentrations of redox pairs could regain full peptide-binding capacity (Figure 4B)

### High-throughput screening assays of peptide-MHC class II binding (ELISA)

We have recently generated a quantitative ELISA assay for measuring peptide-MHC class I interaction [24]. In this assay, titrations of peptide and a fixed low concentration of refolding MHC molecules are co-incubated. The concentrations of the resulting complexes are determined, and it is possible to calculate the K_D _values directly from the saturation curves. An important advantage of this approach is that there is no need to identify a reference peptide for each MHC to be addressed. However, this type of assay relies on the identification of a conformation-dependent MHC class II-specific antibody capable of discriminating between empty and peptide-loaded complexes.

To construct a quantitative sandwich ELISA for HLA-DR molecules, we decided to take advantage of our MHC class II molecules being biotinylated. Streptavidin coated plates were used to capture biotinylated HLA-DR molecules. A panel of anti-MHC class II antibodies were tested as detection antibodies using a refolded and purified biotinylated peptide-DR1 complex as target (additional file 1, Figure 3) The monoclonal anti-DR antibodies LB3.1, D1.12, L243 and G8 all yielded reasonable signals and were subsequently tested for their suitability as a detecting antibody.

**Figure 5:**
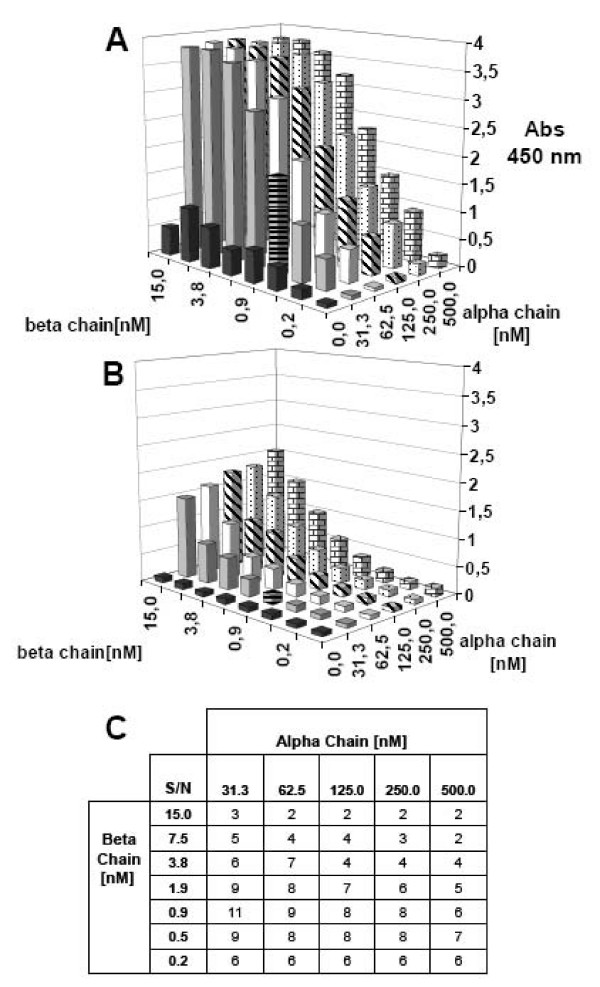
**Checkerboard titrations of DR4 α and β chains analyzed by ELISA**. Various concentrations of urea denatured α and β chains were incubated with (A) and without (B) 2 μM HA_306–318 _in refolding buffer pH 8. After 48 h incubation, the assay was analyzed in the sandwich ELISA assay using streptaviding as capture reagent and L243 as detection antibody. (C) Signal to noise ratios were calculated from samples with and without HA_306–318_. In this case, the combination of 31 nM α chain and 1 nM β chain was determined to be optimal.

Since the final assay would be based upon the correct pairing and refolding of separate MHC class II α and β chains, each potentially refolding with their own efficiency, it was important to determine the optimal concentration of each chain needed to generate peptide receptive complexes. Two-dimensional titrations were performed using pre-oxidized denatured α and β chains diluted to each their specified concentration and incubated in the presence, or absence, of an excess of binding peptide. After 48 h incubation at 18°C, the samples were analyzed in an ELISA using streptavidin as capture reagent, and L243, D1.12, LB3.1, or G8 as detection antibodies. An example of DR4 complex formation analyzed using L243 as the detection antibody is shown in Figure 5. For each of the 48 combinations of MHC class II α and β chains concentrations a signal (+ peptide, Figure 5A) and the corresponding noise (-peptide, Figure 5B) was determined, from these values a signal to noise ratios could be calculated (Figure 5C). In this case, final concentrations of 31 nM α and 1 nM β chains gave the highest signal to noise ratio. In a similar way, optimal concentrations were determined for other DR molecules (data not shown). The four selected antibodies were compared using these optimal MHC class II α and β chain concentrations to generate the different DR complexes in the presence, or absence, of binding peptide. Overall, L243 gave the best signal to noise ratios for the DR proteins tested; D1.12 and LB3.1 gave less discriminatory power; and G8 gave no or very little discriminatory power (the latter is perhaps not surprising as G8 is specific for the superantigen binding site [9]). L243 recognizes an epitope on HLA DR α chain [25] and has previously been used in DR peptide binding assays [8]. In subsequent DR experiments, L243 was used as a detection antibody. In the same manner, suitable antibodies for peptide-dependent DQA1*0501/DQB1*0201 (*in casu *9,3F10) DP401 (*in casu *B7/21) or murine I-E^d ^(*in casu *14.4.4s) complex formation were identified.

In an attempt to reduce the background further, different detergents, a chaotrope (urea) or an elevated temperature, were attempted included in the detection step. Both DR1 and DR4 were tested. In all cases, the inclusion of detergents, urea or an increased temperature, increased the signal to noise ratios; unfortunately at the cost of overall signal. The most pronounced signal to noise improvement (about three fold) was obtained with the mildly denaturing detergent DOC, however, at a concomitant loss of half the signal. None of these manipulations were subsequently used.

**Figure 6:**
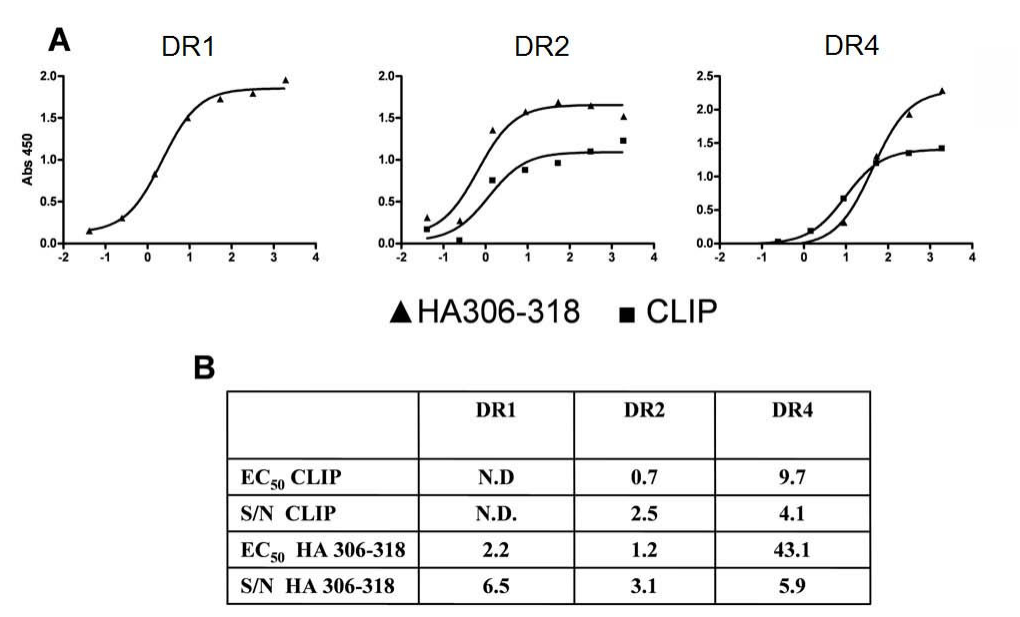
**ELISA-based peptide-MHC class II binding assay**. Urea denatured α and β chains of DR1, DR2 and DR4 were diluted into titrations of CLIP or HA_306–318 _peptides in refolding buffer pH 8 (final concentrations of α and β chains were 31 and 2; 31 and 1; and 31 and 1 nM, respectively). After 24 h incubation at 18°C, the assay was developed as described in materials and methods. The measurements were background subtracted and analysed in Graphpad Prism as previously described. (A) Saturation curve for (black triangle) HA_306–318 _and (black square) CLIP binding to DR1 (left hand panel), DR2 (middle panel) or DR4 (right hand panel) plotted as Absorbance at 450 nm versus the logarithm(10) of the peptide concentration in nM. (B) EC_50 _values and signal to noise ratios (calculated as the ratio between signal at maximum peptide concentration and background). Not determined (N.D.).

Peptide titrations were then carried out on the DR molecules, DR1, DR2a and DR4, using optimized α and β chain concentrations (Figure 6A) followed by the above described L243-driven ELISA analysis, and curve fitting the results. The affinities were approximated from the peptide concentrations leading to half-saturation of the MHC; the EC_50_'s (Figure 6B). The ELISA-based assay appeared capable of measuring peptide-binding affinities for all three DR molecules, and affinities in the low nanomolar range could be determined. From the titration curves it is apparent that the HA_306–318 _peptide gives a higher B_max _values compared to the CLIP peptide 97–120. The reason for this is not known, but could be caused by different complex stabilities affecting the number of complexes surviving the ELISA processing and development procedures.

### High-throughput screening assays of peptide-MHC class II binding (direct LOCI)

**Figure 7:**
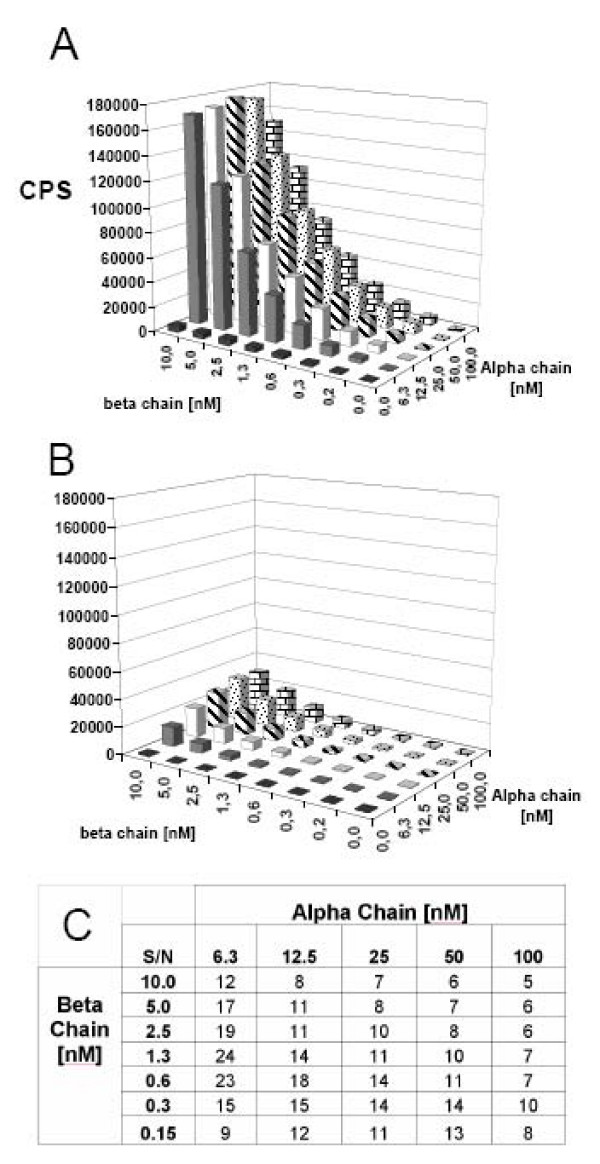
**Checkerboard titrations of DR4 α and β chains analyzed by LOCI**. Various concentrations of urea denatured α and β chains incubated with (A) and without (B) 2 μM HA_306–318 _in refolding buffer pH 8. After 48 h of incubation, the assay was analyzed by LOCI using L243 antibody coupled to acceptor beads and streptavidin coupled donor beads. (C) Signal to noise ratios were calculated from samples with and without HA_306–318_. In this case, concentrations of 6.3 nM α chains and 1.3 nM β chains were determined to be optimal.

We have recently developed a HTS assay for peptide-MHC class I binding using a non-radioactive bead-based homogenous proximity assay: Luminescent Oxygen Channeling Immunoassay [13]. As one tag, we used the biotin group engineered unto the β chain. As the other tag, we used conformation-dependent MHC class II specific antibodies (L243 for DR; 9,3F10 for DQ; B7/21 for DP and 14.4.4s for I-E^d^). As for the development of the ELISA, two-dimensional titrations of MHC class II α and β chain concentrations were performed and diluted into refolding buffer containing, or not containing, an excess concentration of appropriate binding peptide. After 24 h incubation at 18°C, a mixture of streptavidin-donor beads and antibody coupled acceptor beads were added to the reaction wells and analyzed (see Figure 7). It was evident that peptide-MHC class II complexes required both MHC class II α and β chains, and were generated in a strictly peptide-concentration dependent fashion. In this case, optimal α and β chain concentrations were found to be around 6 nM and 1 nM, respectively. Optimal conditions in terms of whether long or short versions of the different molecules should be used, at what concentrations the α and β chain proteins should be used, and the optimal pH were subsequently determined (additional file 1, Table 2 and Figure 4). Strikingly, signal to noise ratios observed with the LOCI technique were consistently better than those observed with the more traditional ELISA technique (e.g. compare Figure 5C with Figure 7C).

**Figure 8:**
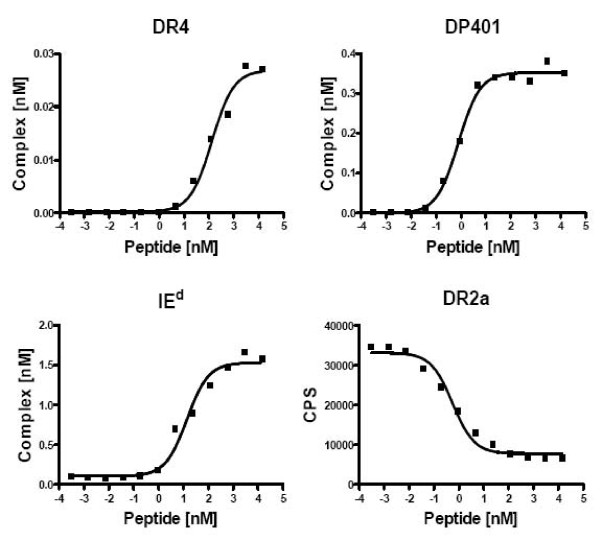
**Direct and competitive LOCI-based peptide-MHC-II binding assay**. Titrations on a promiscuous malaria derived peptide YILLKKILSSRFNQM DR4: K_D _= 133 nM R^2 ^= 0.98. DP401: K_D _= 0.8 nM R^2 ^= 0.99. I-E^d^: K_D _= 14 nM R^2 ^= 0.98. DR2a: inhibition assay, reference peptide 4 nM Biotin-PEG-HA_306–318 _IC_50 _= 0.6 nM R^2 ^= 0.98.

In some cases, poor signal to noise ratios caused by rather high background, compromised the direct binding assay described above. To solve this problem, a competitive assay was developed using a trace concentration of a biotin tagged agonist peptide (*in casu *HA_306–318 _at 4 nm) and non-biotinylated β chains molecules. Complex formation was determined using a LOCI assay detecting the biotinylated peptide with streptavidin donor beads and the peptide-dependent MHC-II conformation with appropriately monoclonal antibody coated acceptor beads. Any test peptide could now be titrated into a binding reaction involving the biotinylated agonist peptide and refolding MHC-II proteins. The resulting inhibition curves could be fitted by non-linear regression allowing the half maximum inhibitory concentration, IC_50_, to be determined (GrapPad prism, see Materials and Methods). Figure 8 shows affinity measurements of a promiscuous malaria-derived peptide binding to four different HLA molecules. To test the direct and competitive assay formats, a panel of peptides was titrated on different MHC II alleles (Table 1). The direct and competitive formats were compared for HLA-DR2a, and found to yield similar results. Note also that known peptide-MHC-II binding interactions reported in the literature could be reproduced [26-34].

## Discussion

A recombinant approach to generate MHC class II reagents would offer many advantages in the analysis of the highly polymorphic MHC class II system. These include ease of production, manipulation, purification, and a high yield at a modest cost. Since the first report of recombinant MHC class II expression was published in 1992, many different approaches to recombinant MHC class II production have been suggested and all of them appear to be in current use i.e. there is no consensus on how to generate recombinant MHC class II molecules. Many variations have been tried. *E. coli *[8,20-22,35-40], insect cells [36,41-62], yeast cells [63-65], and eukaryotic cells [66,67] have all been used as production cells. Natural inter-chain interactions – sometimes including the transmembrane domains [51,52,65-67] sometimes excluding the transmembrane domains (i.e. truncating the chains after the α2 and β2 domains) [20,36,39,40,43,68,69] – have been tried. Alternatively, assembly of MHC α and β chains have been facilitated by phosphatidyl inositol membrane anchoring [61], by fusion to leucine zippers [46,49,50,53,63,70-74], by fusion to IgG chains [42,45,58,75,76], or enforced through the generation of single chain fusion constructs oriented either as α1 β1- or β1α1 [37,38,65]. Molecules have been produced in cells with an intact antigen processing machinery leading to molecules pre-loaded with a collection of naturally offered high-affinity binding peptides [77], or in cells deficient in antigen processing leading to putatively empty molecules. Alternatively, molecules have been loaded with a single predefined high-affinity binding peptide fused to the β chain [43,45-47,49,56-58,64,76,78]. These strategies have aimed at improving one or more aspects of MHC class II production; however, several have involved principles that significantly limit versatility. Systems that require co-expression of α/β heterodimers limit utility since cells expressing each α/β combination of interest will have to be generated one by one. Systems that require linking of a given peptide to the β chain limit utility since the stable binding of the linked peptide compromises subsequent binding experiments and/or replacement with any other peptide. Some reported class II expression systems have had low refolding yields [21,22], some associated binding assays have depended upon a low pH incubation to release endogenous peptides [6,79-83], and others have had to use high concentrations of reporter peptide to detect interaction thus precluding detection of high-affinity interactions [66,73,80-82,84-87].

Protein expression systems based upon *E. coli *expression are potentially fast, versatile and high-yield. Unfortunately, it would appear that many attempts to express class II in E. coli have failed [88]. Major drawbacks of *E. coli *expression include lack of proper folding, disulfide bond formation, and glycosylation leading to aggregate deposition of these non-functional proteins in inclusion bodies. However, a few class II molecules, capable of binding any appropriate peptide offered, have already been successfully produced as isolated subunits in E. coli (DR1 [20,22], DR2a [8] DR4 [89] and I-E^k ^[21]). This demonstrates that it might be possible to express the two chains as isolated subunits and recombine them to generate any desired heterodimer capable of binding any appropriate peptide. This should lead to considerable savings, in particular for DP and DQ molecules, where a limited number of α and β chains can be combined to generate thousands of different receptors. Here, we illustrate this latter point by making HLA-DP and DQ molecules composed of polymorphic α chains (DP1A*0301 and DQA1*0501) paired with the polymorphic β chains (DPB1*0401 and DQB1*0201) respectively.

Here, we have generated an efficient *E. coli *-based expression system for MHC class II molecules. Our approach to E. coli production of MHC class II molecules differs in several respects from those described in the literature. We have used dimerizing modules to facilitate class II α/β pairing and refolding. To the best of our knowledge this has never before been attempted for class II molecules produced in *E. coli *. We have also used a pre-oxidized refolding principle. To our knowledge, all past attempts at producing class II in *E. coli *have involved extraction of class II proteins from inclusion bodies using denaturant solutions containing a reducing agent followed by refolding by dilution into a buffer containing a suitable redox pair to facilitate disulphide bond formation. Such refolding approaches are frequently plagued by low yields. We have successfully produced functional class I molecules in high yield from *E. coli *exploiting the fact that correctly pre-oxidized class I heavy chain molecules can be extracted from inclusion bodies in the absence of reducing agents (note, we don't know whether these disulfide bonds are generated *in vivo *in the bacteria, or *in vitro *during the protein extraction process). Upon dilution into refolding buffer such pre-oxidized class I molecules refolds rapidly and completely. Indeed, our preparations of MHC class II α and β chain proteins contain pre-oxidized species, and they appear essential for the efficiency of the refolding process. We speculate that this is the main reason why all nine MHC class II molecules, that we have produced, have been successfully refolded and been useful in studies involving binding of soluble peptide.

One of our primary motivations to generate recombinant MHC class II molecules is to examine their peptide binding specificity and eventually generate accurate predictors of this event. To this end, biochemical assays should be able to provide large amounts of detailed quantitative binding information. Initially, we developed a standard assay detecting binding of radio-labeled peptide by gel filtration, and used this to systematically vary a number of parameters such as refolding additives, pH, temperature and time etc. A few parameters seemed universally beneficial; most pronounced was the addition of glycerol to the refolding buffer. Stern et al have also noticed this [20]. Other parameters such as length (i.e. where to truncate the α and β chains, additional file 1, Figure 4) and pH were more variable and had to be optimized for each MHC class II heterodimer in question.

Ideally, high-throughput binding assays should be developed to deal with the large number of potential peptide-MHC class II combinations of interest. In the additional information, we demonstrate that a homogenous Scintillation Proximity Assay (SPA) is an attractive high-throughput method for those who consider assays based upon radioactivity as an option. We have also developed assays that do not depend upon radioactivity. One is based on the interaction of the peptide-MHC combination in question followed by a standardized sandwich ELISA to measure the concentration of bound peptide. This can be implemented in most laboratories and yields robust results capable of measuring binding affinities in the low nM range. We have recently developed a non-radioactive, high-throughput homogenous assay for peptide-MHC class I interaction[13]. This assay is based upon Luminescent Oxygen Channeling Immunoassay (LOCI). Here, we demonstrate that this detection mode also works for peptide-MHC class II. In fact, the signal to noise ratio is even better for the LOCI-based assay than it is for the ELISA based assay. The LOCI-based assay has been automated for large-scale screening for MHC class II restricted epitopes (Table 1).

## Conclusion

We have generated a method to produce functional MHC II molecules from pre-oxidized, leucine zipper fused α and β chains individually produced in E. Coli. This protocol is particularly useful when addressing the polymorphic nature of HLA-DP and DQ molecules as demonstrated by the refolding of functional HLA complexes from individually produced α and β chains. Several peptide binding assays were developed including a high-throughput peptide-binding assay based on the homogenous assay platform LOCI. The developed protocols should provide valuable tools to study MHC II function and can be used to generate data for the training of accurate bioinformatics based prediction tools.

## Abbreviations

BSP: biotinylation signal peptide; CTL: cytotoxic T cells; HTS: high throughput screening; MHC-I and -II: major histocompatibility complex class I and II, respectively; DR1: DRA*0101/DRB1*0101; DR2a: DRA*0101/DRB5*0101; DR3: DRA*0101/DRB1*0301; DR4: DRA*0101/DRB1*0401; DRB1*0813: DRA*0101/DRB1*0813; DRB3*0301: DRA*0101/DRB3*0301; DP401: DPA*0301/DPB*0401; TH: T helper cells.

## Competing interests

The authors declare that they have no competing interests.

## Authors' contributions

SJ and SB were involved in the concept and planning of the work and in writing the manuscript. SJ performed most of the experimental work, MH helped with the development of the LOCI assay, KL labelled peptides for the radioactive immuno assay, LLBN cloned all the constructs used for the work.

## Supplementary Material

Additional file 1 **Additional material**. Further information on the following subjects is available as additional material: • Sequences of MHC II constructs used in this work (Figure 1); • Effect of refolding additives (Table 1); • Development of a high throughput scintillation proximity assay (SPA) peptide binding assay (Figure 2); • Selection of DR specific antibodies (Figure 3); • Truncation of DR α and β chains (Figure 4); • The use of the nucleotide exchange factor (GrpE) as a novel MHC II dimerization motif; and • Summary of LOCI assay conditions (Table 2). Click here for file
