# Supplementary material for: Functional recombinant MHC class II molecules and high-throughput peptide-binding assays
Source: Immunome Res. 2009 May 5;5:2. doi: 10.1186/1745-7580-5-2 (PMC2690590; doi:10.1186/1745-7580-5-2)
Supplement: Additional file 1 — Additional material. Further information on the following subjects is available as additional material: • Sequences of MHC II constructs used in this work (Figure 1); • Effect of refolding additives (Table 1); • Development of a high throughput scintillation proximity assay (SPA) peptide binding assay (Figure 2); • Selection of DR specific antibodies (Figure 3); • Truncation of DR α and β chains (Figure 4); • The use of the nucleotide exchange factor (GrpE) as a novel MHC II dimerization motif; and • Summary of LOCI assay conditions (Table 2). [file 1745-7580-5-2-S1.doc]

# Additional Material

Supplementary figure 1. Sequences of MHC II chains used in this work.

Bold underscored: Mutations, Bold: Linker sequences, Underscored: Leucine zipper sequences, Bold italic: GrpE_33-197_, Grey: Natural histidine affinity tag (HAT), Italic: Biotinylation signal peptide (BSP), (Note, the BSP sequence used here is a variant of the GGGLNDIFEA QKIEWHE published by Schatz et al. (1)

**Alpha Chains**

DRA*0101_1-181_LZFos

MIKEEHVIIQ AEFYLNPDQS GEFMFDFDGD EIFHVDMAKK ETVWRLEEFG RFASFEAQGA LANIAVDKAN LEIMTKRSNY TPITNVPPEV TVLTNSPVEL REPNVLICFI DKFTPPVVNV TWLRNGKPVT TGVSETVFLP REDHLFRKFH YLPFLPSTED VYDCRVEHWG LDEPLLKHWE FD**GGGGG**LTD TLQAETDQLE DEKSALQTEI ANLLKEKEKL EFILAA

DRA*0101_1-191_LZFos

MIKEEHVIIQ AEFYLNPDQS GEFMFDFDGD EIFHVDMAKK ETVWRLEEFG RFASFEAQGA LANIAVDKAN LEIMTKRSNY TPITNVPPEV

TVLTNSPVEL REPNVLICFI DKFTPPVVNV TWLRNGKPVT TGVSETVFLP REDHLFRKFH YLPFLPSTED VYDCRVEHWG LDEPLLKHWE

FDAPSPLPET TE**GGGGG**LTD TLQAETDQLE DEKSALQTEI ANLLKEKEKL EFILAA

DRA*0101_1-181_GrpE

MIKEEHVIIQ AEFYLNPDQS GEFMFDFDGD EIFHVDMAKK ETVWRLEEFG RFASFEAQGA LANIAVDKAN LEIMTKRSNY TPITNVPPEV TVLTNSPVEL REPNVLICFI DKFTPPVVNV TWLRNGKPVT TGVSETVFLP REDHLFRKFH YLPFLPSTED VYDCRVEHWG LDEPLLKHWE FDA**GGGGGG*A EQVDPRDEKI ANLEAQLAEA QTRERDGILR VKAEMENLRR RTELDIEKAH KFALEKFINE LLPVIDSLDR ALEVADKANP DMSAMVEGIE LTLKSMLDVV RKFGVEVIAE TNVPLDPNVH QAIAMVESDD VAPGNVLGIM QKGYTLNGRT IRAAMVTVAK***

***AKA***

DPA1*0103_1-181_(C123S)LZFos

MIKADHVSTY AAFVQTHRPT GEFMFEFDED EMFYVDLDKK ETVWHLEEFG QAFSFEAQGG LANIAILNNN LNTLIQRSNH TQATNDPPEV TVFPKEPVEL GQPNTLICHI DKFFPPVLNV TWL**S**NGELVT EGVAESLFLP RTDYSFHKFH YLTFVPSAED FYDCRVEHWG LDQPLLKHWE AQ**GGGG**LTDT LQAETDQLED EKSALQTEIA NLLKEKEKLE FILAA

HAT-DQA1*0501_1-181_(C47S)LZFos

MGKDHLIHNV HKEEHAHAHN KGSIEGRMSE DIVADHVASY GVNLYQSYGP SGQYTHEFDG DEQFYVDLGR KETVW**S**LPVL RQFRFDPQFA LTNIAVLKHN LNSLIKRSNS TAATNEVPEV TVFSKSPVTL GQPNILICLV DNIFPPVVNI TWLSNGHSVT EGVSETSFLS KSDHSFFKIS YLTLLPSAEE SYDCKVEHWG LDKPLLKHWE PEGGGGLTDT LQAETDQLED EKSALQTEIA NLLKEKEKLE FILAA

I-E^d^A_1-191_LZFos

MIKEEHTIIQ AEFYLLPDKR GEFMFDFDGD EIFHVDIEKS ETIWRLEEFA KFASFEAQGA LANIAVDKAN LDVMKERSNN TPDANVAPEV TVLSRSPVNL GEPNILICFI DKFSPPVVNV TWLRNGRPVT EGVSETVFLP RDDHLFRKFH YLTFLPSTDD FYDCEVDHWG LEEPLRKTWE FEEKTLLPET KEN**GGGG**LTD TLQAETDQLE DEKSALQTEI ANLLKEKEKL EFILAA

**Beta chains**

**DR1**

DRB1*0101_1-190_(C30S)LZJunBSP

MGDTRPRFLW QLKFECHFFN GTERVRLLER **S**IYNQEESVR FDSDVGEYRA VTELGRPDAE YWNSQKDLLE QRRAAVDTYC RHNYGVGESF TVQRRVEPKV TVYPSKTQPL QHHNLLVCSV SGFYPGSIEV RWFRNGQEEK AGVVSTGLIQ NGDWTFQTLV MLETVPRSGE VYTCQVEHPS VTSPLTVEWR A**GGGGG**RIAR LEEKVKTLKA QNSELASTAN MLREQVAQLK QKVMNHS**GGG** *LGGLNDIFEA QKIEWHN*

DRB1*0101_1-198_(C30S)LZJunBSP

MGDTRPRFLW QLKFECHFFN GTERVRLLER **S**IYNQEESVR FDSDVGEYRA VTELGRPDAE YWNSQKDLLE QRRAAVDTYC RHNYGVGESF TVQRRVEPKV TVYPSKTQPL QHHNLLVCSV SGFYPGSIEV RWFRNGQEEK AGVVSTGLIQ NGDWTFQTLV MLETVPRSGE VYTCQVEHPS VTSPLTVEWR ARSESAQSK**G GGGG**RIARLE EKVKTLKAQN SELASTANML REQVAQLKQK VMNHS**GGG***LG GLNDIFEAQK IEWHN*

DRB1*0101_1-190_(C30S)GrpE

MGDTRPRFLW QLKFECHFFN GTERVRLLER **S**IYNQEESVR FDSDVGEYRA VTELGRPDAE YWNSQKDLLE QRRAAVDTYC RHNYGVGESF TVQRRVEPKV TVYPSKTQPL QHHNLLVCSV SGFYPGSIEV RWFRNGQEEK AGVVSTGLIQ NGDWTFQTLV MLETVPRSGE VYTCQVEHPS VTSPLTVEWR A**GGGGGG*AEQ VDPRDEKIAN LEAQLAEAQT RERDGILRVK AEMENLRRRT ELDIEKAHKF ALEKFINELL PVIDSLDRAL EVADKANPDM SAMVEGIELT LKSMLDVVRK FGVEVIAETN VPLDPNVHQA IAMVESDDVA PGNVLGIMQK GYTLNGRTIR AAMVTVAKAK A***

**DR3**

DRB1*0301_1-190_LZJunBSP

MGDTRPRFLE YSTSECHFFN GTERVRYLDR YFHNQEENVR FDSDVGEFRA VTELGRPDAE YWNSQKDLLE QKRGRVDNYC RHNYGVVESF TVQRRVHPKV TVYPSKTQPL QHHNLLVCSV SGFYPGSIEV RWFRNGQEEK TGVVSTGLIH NGDWTFQTLV MLETVPRSGE VYTCQVEHPS VTSPLTVEWR A**GGGGG**RIAR LEEKVKTLKA QNSELASTAN MLREQVAQLK QKVMNHS**GGG** *LGGLNDIFEA QKIEWHN*

**DR4**

DRB1*0401_1-190_LZJunBSP

MGDTRPRFLE QVKHECHFFN GTERVRFLDR YFYHQEEYVR FDSDVGEYRA VTELGRPDAE YWNSQKDLLE QKRAAVDTYC RHNYGVGESF TVQRRVYPEV TVYPAKTQPL QHHNLLVCSV NGFYPGSIEV RWFRNGQEEK TGVVSTGLIQ NGDWTFQTLV MLETVPRSGE VYTCQVEHPS LTSPLTVEWR A**GGGGG**RIAR LEEKVKTLKA QNSELASTAN MLREQVAQLK QKVMNHS**GGG** *LGGLNDIFEA QKIEWHN*

DRB1*0401_1-198_LZJunBSP

MGDTRPRFLE QVKHECHFFN GTERVRFLDR YFYHQEEYVR FDSDVGEYRA VTELGRPDAE YWNSQKDLLE QKRAAVDTYC RHNYGVGESF TVQRRVYPEV TVYPAKTQPL QHHNLLVCSV NGFYPGSIEV RWFRNGQEEK TGVVSTGLIQ NGDWTFQTLV MLETVPRSGE VYTCQVEHPS LTSPLTVEWR ARSESAQSK**G GGGG**RIARLE EKVKTLKAQN SELASTANML REQVAQLKQK VMNHS**GGG***LG GLNDIFEAQK IEWHN*

**DRB1*0813**

DRB1*0813_1-190_LZJunBSP

MGDTRPRFLE YSTGECYFFN GTERVRFLDR YFYNQEEYVR FDSDVGEYRA VTELGRPDAE YWNSQKDLLE DRRALVDTYC RHNYGVGESF TVQRRVHPKV TVYPSKTQPL QHHNLLVCSV SGFYPGSIEV RWFRNGQEEK TGVVSTGLIH NGDWTFQTLV MLETVPRSGE VYTCQVEHPS VTSPLTVEWS A**GGGGG**RIAR LEEKVKTLKA QNSELASTAN MLREQVAQLK QKVMNHS**GGG** *LGGLNDIFEA QKIEWHN*

**DRB3*0301**

DRB3*0301_1-190_LZJunBSP

MGDTRPRFLE LLKSECHFFN GTERVRFLER YFHNQEEFVR FDSDVGEYRA VTELGRPVAE SWNSQKDLLE QKRGQVDNYC RHNYGVVESF TVQRRVHPQV TVYPAKTQPL QHHNLLVCSV SGFYPGSIEV RWFRNGQEEK TGVVSTGLIH NGDWTFQTLV MLETVPRSGE VYTCQVEHPS VTSPLTVEWR AGGGGGRIAR LEEKVKTLKA QNSELASTAN MLREQVAQLK QKVMNHSGGG *LGGLNDIFEA QKIEWHE*

**DR2a**

DRB5*0101_1-190_LZJunBSP

MGDTRPRFLQ QDKYECHFFN GTERVRFLHR DIYNQEEDLR FDSDVGEYRA VTELGRPDAE YWNSQKDFLE DRRAAVDTYC RHNYGVGESF TVQRRVEPKV TVYPARTQTL QHHNLLVCSV NGFYPGSIEV RWFRNSQEEK AGVVSTGLIQ NGDWTFQTLV MLETVPRSGE VYTCQVEHPS VTSPLTVEWR A**GGGGG**RIAR LEEKVKTLKA QNSELASTAN MLREQVAQLK QKVMNHS**GGG** *LGGLNDIFEA QKIEWHN*

DRB5*0101_1-198_LZJunBSP

MGDTRPRFLQ QDKYECHFFN GTERVRFLHR DIYNQEEDLR FDSDVGEYRA VTELGRPDAE YWNSQKDFLE DRRAAVDTYC RHNYGVGESF TVQRRVEPKV TVYPARTQTL QHHNLLVCSV NGFYPGSIEV RWFRNSQEEK AGVVSTGLIQ NGDWTFQTLV MLETVPRSGE VYTCQVEHPS VTSPLTVEWR AQSESAQSK**G GGGG**RIARLE EKVKTLKAQN SELASTANML REQVAQLKQK VMNHS**GGG***LG GLNDIFEAQK IEWHN*

**DP401**

DPB1*0401_1-190_LZJunBSP

MRATPENYLF QGRQECYAFN GTQRFLERYI YNREEFARFD SDVGEFRAVT ELGRPAAEYW NSQKDILEEK RAVPDRMCRH NYELGGPMTL QRRVQPRVNV SPSKKGPLQH HNLLVCHVTD FYPGSIQVRW FLNGQEETAG VVSTNLIRNG DWTFQILVML EMTPQQGDVY TCQVEHTSLD SPVTVEWKA**G GGGG**RIARLE EKVKTLKAQN SELASTANML REQVAQLKQK VMNHS**GGG***LG GLNDIFEAQK IEWHN*

**DQA1*0501/DQB1*0201**

DQB1*0201LZ_1-190_JunBSP

MRDSPEDFVY QFKGMCYFTN GTERVRLVSR SIYNREEIVR FDSDVGEFRA VTLLGLPAAE YWNSQKDILE RKRAAVDRVC RHNYQLELRT TLQRRVEPTV TISPSRTEAL NHHNLLVCSV TDFYPAQIKV RWFRNDQEET AGVVSTPLIR NGDWTFQILV MLEMTPQRGD VYTCHVEHPS LQSPITVEWG GGGGRIARLE EKVKTLKAQN SELASTANML REQVAQLKQK VMNHSGGGGG G*LNDIFEAQK IEWHN*

**I-E^d^**

I-E^d^B_1-198_LZJunBSP

MVRDTRPRFL EYVTSECHFY NGTQHVRFLE RFIYNREENL RFDSDVGEYR AVTELGRPDA ENWNSQPEIL EDARASVDTY CRHNYEISDK FLVRRRVEPT VTVYPTKTQP LEHHNLLVCS VSDFYPGNIE VRWFRNGKEE ETGIVSTGLV RNGDWTFQTL VMLETVPQSG EVYTCQVEHP SLTDPVTVEW KAQSTSAQNK **GGGGG**RIARL EEKVKTLKAQ NSELASTANM LREQVAQLKQ KVMNHS**GGG***L GGLNDIFEAQ KIEWHN*

| **Compound** | **Harmfull** |  |
| --- | --- | --- |
| Isopropanol | Yes | 50 % signal reduction at 5 Vol % |
| 2-Butanol | Yes | 50 % signal reduction at 2.5 Vol % |
| N-Laurylsarcosin | Yes | 50 % signal reduction at 0.01 Vol % |
| NP40 | Yes | 50 % signal reduction at 0.01 Vol % |
| parachlorophenol (pCP) | Yes | 50 % signal reduction at 4 mM |
| NaCl | Yes | 50 % signal reduction at 100 mM |
| DOC | Yes/No | Positive effect up to 0.02 % Harmfull at higher concentrations |
| Urea | Yes/No | Ok up to 400 mM. Harmfull at higher concentrations |
| Pluriol F68 | No | Ok up to 0.4 w/w % |
| Dextran | No | Ok up to 0.5 w/w % |
| PEG6000/20000 | No | Ok up to 0.5 w/w % |
| Tween20 | No | Ok up to 0.5 Vol % |
| Beta octyl glucoside | No | Ok up to 0.5 w/w % |
| Glycerol | No | Enhances folding in whole range tested |

Table 1 Qualitative effects of additives on the refolding of DR1. Additives where titrated in refolding buffer pH 8 (without glycerol) and supplemented with 3nM ^125^I labeled YHA306-318 and 80 nM urea denatured DR1 α and β chains. Following incubation the experiments were analyzed by the spun column assay as described in materials and methods.

### MHC class II binding measured by high-throughput scintillation proximity assay (SPA)

We wanted to exploit the fact that we have biotinylated MHC class II molecules and a radioactively labeled reference peptides capable of binding to the same MHC molecule with high affinity. This should enable a scintillation proximity assay (SPA), which is a bead-based assay relying on the emission of beta particles in the vicinity (within approximately 10 µm for ^125^I) of a bead composed of a scintillant (polyvinyltoluene) and a capturing outer layer. Since no washing step is needed prior to signal generation, this assay is homogenous and highly suitable for high throughput screening (HTS) (2). Using the previously defined optimal conditions, denatured DRA*0101 and DRB1*0101 chains were diluted to final equimolar concentrations of 30nM into a refolding buffer containing 2 nM ^125^I labeled YHA_306-318_ peptide and a dose-titration of competing test peptide. The reaction mixtures were incubated for 24h at 18^o^C. Subsequently, 50µl reaction mixture was combined with 100µl of a solution of streptavidin coated SPA beads (1.3 mg/ml in PBS, GE healthcare) in white 96-well OptiPlates (Perkin Elmer), incubated for 2h at RT and finally read on a TopCount reader (Perkin Elmer) according to the manufacturers’ recommendations. The GraphPad program, Prism, was used to analyze data after fitting non-linearly to a sigmoid dose response curve (Supplementary Figure 2) leading to regression coefficients over 0.99, a high signal to noise ratio and IC_50_ values in the low nanomolar area. Compared to the spun column assay, the SPA assay qualitatively revealed the same MHC class II specificity, and quantitatively measured the affinities of the same peptide-MHC class II combinations to be slightly better. The latter may be a result of the SPA being a homogenous assay is less susceptible to any complex instability.

Supplementary Figure 2 Scintillation proximity assay (SPA). 30 nM urea denatured DR1 α and β chain molecules were diluted into refolding buffer pH 8 supplemented with 2nM ^125^I labeled YHA_306-318_ and titrations of competitor peptides or proteins as indicated. After incubation, reaction mixture was combined with a solution of streptavidin coated SPA beads and read on a TopCount reader (Perkin Elmer). (A) Results were plotted as cpm versus Log10 to the inhibitor concentration in nM, and analyzed using Prism as previously described. The curves yielded regression coefficients over 0.99, a high signal to noise ratio and IC_50_ values in the low nanomolar area. Invariant chain, HA_306-318H6_ and a histidine tagged CLIP peptide was demonstrated to be good binders whereas the invariant chain fragment 118-208 (3) was a poor binder.

### Selection of DR specific antibody


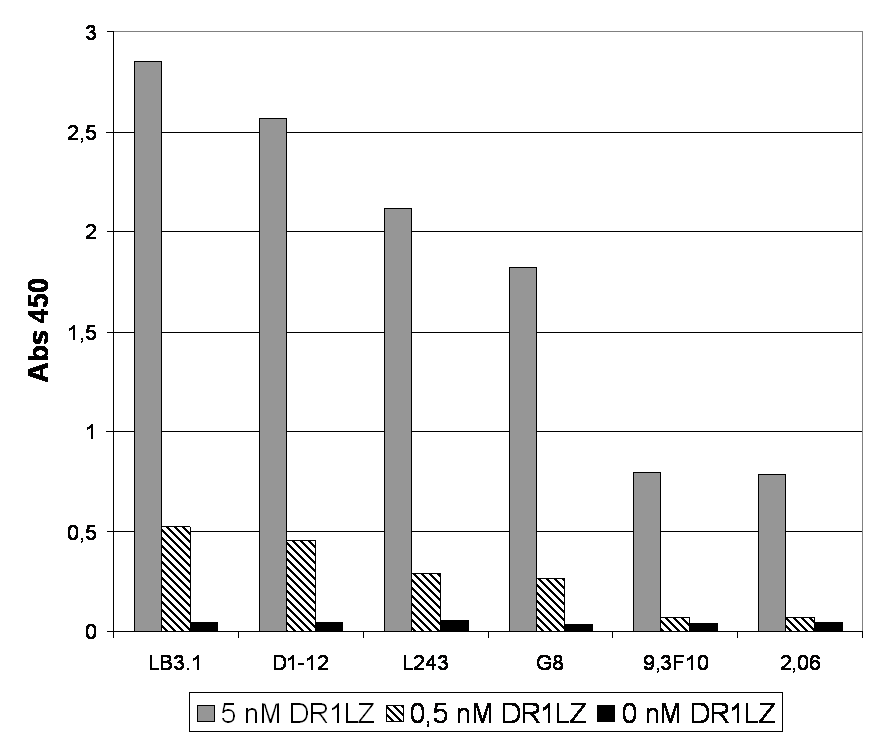


Supplementary Figure 3 ELISA selection of a DR specific antibody using a purified DR1 standard. Dilutions of a purified biotinylated DR1 complex in PBS were added to a streptavidin plate. After incubation and washing a panel of detecting antibodies were added (10 µg/ml, LB3.1, D1.12, L243, G8, 9.3F10 and 2.06). The plates were washed and developed as described in materials and methods.

### Truncation of DR α and β chains

Soluble expression of recombinant membrane anchored proteins often relies on the proper truncation of the expressed construct. Precise definition of boundaries is important, and variation by two or three residues can alter the behavior of the protein (4). Investigation of crystal structures of 6 different MHC II molecules covering the following alleles I-A^k^ *(5,* 6) I-E^k^ (7), DR1 (8), DR4 (9) and DQ8 *(*10) revealed that a defined crystal structure, seemed to end at consensus positions 181/182 for α chain and at position 190 for β chain. This is in contrast to the native protein, where the transmembrane segment starts at residue 192 and 199 for alpha and beta chains, respectively. We therefore produced DR1, DR2a and DR4 α and β chains as long and short truncations, the chains were combined and refolded in the presence or absence of HA_306-318_, the results indicated that chain length affected refolding efficiency dramatically (Supplementary Figure 4). Unfortunately, an optimal length was difficult to find, but the data suggest that truncation may be a contributing factor in the successful expression of MHC II molecules.

**CPS**

Supplementary Figure 4 Comparison of short and long truncations of alpha and beta chains. Experiments were done in duplicate and standard deviations are shown. Stocks of urea denatured MHC were diluted into refolding buffer with (black bar) and without (grey bar, background) 2 µM HA306-318 and incubated at pH 7 for 24h at 18 ºC. Final concentrations were DR1: 6.5 / 2.5, DR2: 6.5 / 1.3 and DR4: 6.5 / 1.3 (α nM/ β nM, respectively). The combinations of long (L) and short (S) chains are shown as: length of α chain/length of β chain. For example, L/L comprises residue 1-191 of α chain and 1-198 of β chain. Following incubation, the experiment was analyzed by the LOCI assay as described in materials and methods.

### A novel MHC II dimerization motif

Leucine zippers are widely used to restore the stabilizing and dimerizing effects of the two transmembrane segments, that are lost upon truncation of the α and β chain *(11-*20).

The dimerizing HSP 70 co-chaperone GrpE was introduced as a fusion tag by Davis *et al* *(*21), to increase solubility of heterologously produced proteins in *E. coli*. For this project however, its preferential existence as a dimer was also important *(22-*24). We produced the DR1 allele as a construct, comprising the short truncations of the α (1-181) and β chain (1-190), fused C terminally to GrpE (amino acids 33-197). At the C terminal of the beta chain, the natural histidine tag sequence (HAT) was added to facilitate purification *(*25).

In initial experiments, using Iodine labeled YHA_306-318_ and spun column assay, the GrpE and leucine zipper constructs yielded indistinguishable results, and it was concluded that the specificity was not compromised by GrpE (data not shown). The construct was not biotinylated and subsequently used in the competitive LOCI, described below.

### LOCI Assay conditions

| **Allele** | **Alpha chain** | **Beta Chain** | **Conc α / β (nM)** | **Assay type** | **pH** | **antibody** |
| --- | --- | --- | --- | --- | --- | --- |
| DR1GrpE | DRA*0101_1-181_GrpE | DRB1*0101_1-190_(C30S)GrpE | 5 / 5 | Competitive | 6-7.5 | L243 |
| DR1 | DRA*0101_1-191_ | DRB1*0101_1-198_(C30S) | 7 / 3 | Direct | 6 | L243 |
| DR2a | DRA*0101_1-181_ | DRB5*0101_1-190_ (no biotin) | 13 / 5 | Competitive | 6-7.5 | L243 |
| DR2a | DRA*0101_1-181_ | DRB5*0101_1-190_ | 6 / 1 | Direct | 6 | L243 |
| DR3 | DRA*0101_1-191_ | DRB1*0301_1-190_ | 6 / 25 | Direct | 6-7 | L243 |
| DR4 | DRA*0101_1-181_ | DRB1*0401_1-190_ | 13 / 3 | Direct | 6-7.5 | L243 |
| DRB1*0813 | DRA*0101_1-181_ | DRB1*0813_1-190_ | 10 / 10 | Direct | 6-7.5 | L243 |
| DRB3*0301 | DRA*0101_1-181_ | DRB3*0301_1-190_ | 6/12 | Direct | 7 | L243 |
| DQA1*0501/DQB1*0201 | HAT-DQA1*0501_1-181_(C47S) | DQB1*0201LZ_1-190_ | 11/11 | Direct | 7 | 9,3F10 |
| DP401 | DPA1*0103(c123s)_1-181_ | DPB1*0401_1-190_ | 13 / 6 | Direct | 7 | B7/21 |
| I-E^d^ | I-E^d^A_1-191_ | I-E^d^B_1-198_ | 13 / 13 | Direct | 6 + 7.5 | 14.4.4s |

Table 2 Summary of assay conditions in peptide binding assays.

The pH range, in which the assay can be conducted with a signal to ratio better than 5 is indicated.

(1) Schatz, P. J. (1993) Use of peptide libraries to map the substrate specificity of a peptide-modifying enzyme: a 13 residue consensus peptide specifies biotinylation in Escherichia coli. *Biotechnology (N Y)* *11*, 1138-43.

(2) Wu, S., and Liu, B. (2005) Application of scintillation proximity assay in drug discovery. *BioDrugs* *19*, 383-92.

(3) Park, S. J., Sadegh-Nasseri, S., and Wiley, D. C. (1995) Invariant chain made in Escherichia coli has an exposed N-terminal segment that blocks antigen binding to HLA-DR1 and a trimeric C-terminal segment that binds empty HLA-DR1. *Proc Natl Acad Sci U S A* *92*, 11289-93.

(4) Reich, S., Puckey, L. H., Cheetham, C. L., Harris, R., Ali, A. A., Bhattacharyya, U., Maclagan, K., Powell, K. A., Prodromou, C., Pearl, L. H., Driscoll, P. C., and Savva, R. (2006) Combinatorial Domain Hunting: An effective approach for the identification of soluble protein domains adaptable to high-throughput applications. *Protein Sci* *15*, 2356-65.

(5) Reinherz, E. L., Tan, K., Tang, L., Kern, P., Liu, J., Xiong, Y., Hussey, R. E., Smolyar, A., Hare, B., Zhang, R., Joachimiak, A., Chang, H. C., Wagner, G., and Wang, J. (1999) The crystal structure of a T cell receptor in complex with peptide and MHC class II. *Science* *286*, 1913-21.

(6) Fremont, D. H., Monnaie, D., Nelson, C. A., Hendrickson, W. A., and Unanue, E. R. (1998) Crystal structure of I-Ak in complex with a dominant epitope of lysozyme. *Immunity* *8*, 305-17.

(7) Wilson, N., Fremont, D., Marrack, P., and Kappler, J. (2001) Mutations changing the kinetics of class II MHC peptide exchange. *Immunity* *14*, 513-22.

(8) Stern, L. J., Brown, J. H., Jardetzky, T. S., Gorga, J. C., Urban, R. G., Strominger, J. L., and Wiley, D. C. (1994) Crystal structure of the human class II MHC protein HLA-DR1 complexed with an influenza virus peptide. *Nature* *368*, 215-21.

(9) Bolin, D. R., Swain, A. L., Sarabu, R., Berthel, S. J., Gillespie, P., Huby, N. J., Makofske, R., Orzechowski, L., Perrotta, A., Toth, K., Cooper, J. P., Jiang, N., Falcioni, F., Campbell, R., Cox, D., Gaizband, D., Belunis, C. J., Vidovic, D., Ito, K., Crowther, R., Kammlott, U., Zhang, X., Palermo, R., Weber, D., Guenot, J., Nagy, Z., and Olson, G. L. (2000) Peptide and peptide mimetic inhibitors of antigen presentation by HLA-DR class II MHC molecules. Design, structure-activity relationships, and X-ray crystal structures. *J Med Chem* *43*, 2135-48.

(10) Lee, K. H., Wucherpfennig, K. W., and Wiley, D. C. (2001) Structure of a human insulin peptide-HLA-DQ8 complex and susceptibility to type 1 diabetes. *Nat Immunol* *2*, 501-7.

(11) Yang, J., Jaramillo, A., Shi, R., Kwok, W. W., and Mohanakumar, T. (2004) In vivo biotinylation of the major histocompatibility complex (MHC) class II/peptide complex by coexpression of BirA enzyme for the generation of MHC class II/tetramers. *Hum Immunol* *65*, 692-9.

(12) Huang, J. C., Vestberg, M., Minguela, A., Holmdahl, R., and Ward, E. S. (2004) Analysis of autoreactive T cells associated with murine collagen-induced arthritis using peptide-MHC multimers. *Int Immunol* *16*, 283-93.

(13) Quarsten, H., McAdam, S. N., Jensen, T., Arentz-Hansen, H., Molberg, O., Lundin, K. E., and Sollid, L. M. (2001) Staining of celiac disease-relevant T cells by peptide-DQ2 multimers. *J Immunol* *167*, 4861-8.

(14) Kalandadze, A., Galleno, M., Foncerrada, L., Strominger, J. L., and Wucherpfennig, K. W. (1996) Expression of recombinant HLA-DR2 molecules. Replacement of the hydrophobic transmembrane region by a leucine zipper dimerization motif allows the assembly and secretion of soluble DR alpha beta heterodimers. *J Biol Chem* *271*, 20156-62.

(15) Gauthier, L., Smith, K. J., Pyrdol, J., Kalandadze, A., Strominger, J. L., Wiley, D. C., and Wucherpfennig, K. W. (1998) Expression and crystallization of the complex of HLA-DR2 (DRA, DRB1*1501) and an immunodominant peptide of human myelin basic protein. *Proc Natl Acad Sci U S A* *95*, 11828-33.

(16) Novak, E. J., Liu, A. W., Nepom, G. T., and Kwok, W. W. (1999) MHC class II tetramers identify peptide-specific human CD4(+) T cells proliferating in response to influenza A antigen. *J Clin Invest* *104*, R63-7.

(17) Reichstetter, S., Ettinger, R. A., Liu, A. W., Gebe, J. A., Nepom, G. T., and Kwok, W. W. (2000) Distinct T cell interactions with HLA class II tetramers characterize a spectrum of TCR affinities in the human antigen-specific T cell response. *J Immunol* *165*, 6994-8.

(18) Kwok, W. W., Liu, A. W., Novak, E. J., Gebe, J. A., Ettinger, R. A., Nepom, G. T., Reymond, S. N., and Koelle, D. M. (2000) HLA-DQ tetramers identify epitope-specific T cells in peripheral blood of herpes simplex virus type 2-infected individuals: direct detection of immunodominant antigen-responsive cells. *J Immunol* *164*, 4244-9.

(19) Bioley, G., Jandus, C., Tuyaerts, S., Rimoldi, D., Kwok, W. W., Speiser, D. E., Tiercy, J. M., Thielemans, K., Cerottini, J. C., and Romero, P. (2006) Melan-A/MART-1-Specific CD4 T Cells in Melanoma Patients: Identification of New Epitopes and Ex Vivo Visualization of Specific T Cells by MHC Class II Tetramers. *J Immunol* *177*, 6769-79.

(20) Yang, J., Huston, L., Berger, D., Danke, N. A., Liu, A. W., Disis, M. L., and Kwok, W. W. (2005) Expression of HLA-DP0401 molecules for identification of DP0401 restricted antigen specific T cells. *J Clin Immunol* *25*, 428-36.

(21) Davis, G. D., Elisee, C., Newham, D. M., and Harrison, R. G. (1999) New fusion protein systems designed to give soluble expression in Escherichia coli. *Biotechnol Bioeng* *65*, 382-8.

(22) Harrison, C. J., Hayer-Hartl, M., Di Liberto, M., Hartl, F., and Kuriyan, J. (1997) Crystal structure of the nucleotide exchange factor GrpE bound to the ATPase domain of the molecular chaperone DnaK. *Science* *276*, 431-5.

(23) Mehl, A. F., Heskett, L. D., and Neal, K. M. (2001) A GrpE mutant containing the NH(2)-terminal "tail" region is able to displace bound polypeptide substrate from DnaK. *Biochem Biophys Res Commun* *282*, 562-9.

(24) Schonfeld, H. J., Schmidt, D., Schroder, H., and Bukau, B. (1995) The DnaK chaperone system of Escherichia coli: quaternary structures and interactions of the DnaK and GrpE components. *J Biol Chem* *270*, 2183-9.

(25) Patwardhan, A. V., Goud, G. N., Koepsel, R. R., and Ataai, M. M. (1997) Selection of optimum affinity tags from a phage-displayed peptide library. Application to immobilized copper(II) affinity chromatography. *J Chromatogr A* *787*, 91-100.
